# Positive attentional bias mediates the relationship between trait emotional intelligence and trait affect

**DOI:** 10.1038/s41598-022-25317-9

**Published:** 2022-12-01

**Authors:** Thomas Suslow, Dennis Hoepfel, Vivien Günther, Anette Kersting, Charlott Maria Bodenschatz

**Affiliations:** Department of Psychosomatic Medicine and Psychotherapy, University of Leipzig Medical Center, Semmelweisstr. 10, 04103 Leipzig, Germany

**Keywords:** Psychology, Risk factors

## Abstract

Emotional intelligence and, in particular, the component emotion regulation may increase well-being and improve mood and coping with negative emotions. In the present eye-tracking study, we examined whether attention allocation to positive stimuli mediates the relationship between emotion regulation abilities and trait affect. Gaze behavior of 104 healthy adults was analyzed in a free-viewing task, in which happy, sad, angry, and neutral faces were shown simultaneously for ten seconds. Dwell time on facial expressions was used as indicator of attention allocation. Trait emotional intelligence was assessed using the Self-Rated Emotional Intelligence Scale. Self-report measures of state and trait positive and negative affect, trait anxiety, and depression were administered. In general, participants viewed longer at happy than at negative or neutral faces. The results of mediation analyses indicated that intrapersonal and interpersonal emotion regulation abilities were indirectly related to trait positive affect through attention to happy faces. Moreover, dwell time on happy faces had a mediating effect on the relationship between interpersonal emotion regulation ability and trait anxiety. Preference for positive social signals might form one attentional pathway through which emotion regulation abilities promote positive mood and buffer the development of anxiety reactions in everyday life.

## Introduction

Emotional intelligence is a psychological construct that emerged and popularized in the late twentieth century^1,2^. A very influential definition of emotional intelligence proposed by Mayer and Salovey^3^ comprises four related components that are assumed to be hierarchically ordered from rather elemental to more complex abilities: (a) the ability to accurately perceive emotions in oneself and others, as well as in other environmental stimuli, (b) the ability to generate and access emotions to assist reasoning and problem-solving, (c) the ability to understand emotional reactions, their verbalization, causes, and consequences, and (d) the ability to reduce, enhance, and modify emotional reactions in oneself and other persons^4^. The abilities to perceive and use emotions have been subsumed under the term experiential facet of emotional intelligence, which are considered to reflect lower-order or rapid abilities in emotional processing^5,6^. Furthermore, the abilities to understand and regulate emotions have been summarized under strategic emotional intelligence, which is assumed to comprise higher-order or deliberate processes of emotional reasoning and control^5,6^.

In the last decades, various theoretical models of emotional intelligence have been developed that can be divided into three main groups according to the type of test instrument administered to assess the construct: performance-based ability, self-report trait, and self-report mixed models^7^. Ability models define emotional intelligence as a type of intelligence or aptitude and use performance tasks that require knowledge and processing of emotional information to solve emotion-related problems^8,9^. Ability-based measures give a good indication of individuals’ objective ability to understand and process emotions and are tests of maximal performance. Trait models conceive emotional intelligence as typical behavioral tendencies and self-perceptions concerning one’s ability to recognize and utilize emotional information^10–12^. Trait emotional intelligence is measured with self-report questionnaires. Self-report mixed models also assess emotional intelligence through self-report instruments, but their construct is broader and includes additionally, for example, motivations, attitudes, social skills, and competencies^13^.

Emotional intelligence has been found to be associated with mental and physical health^14^, subjective well-being, optimism, and happiness^15–17^. Emotional intelligence seems to operate as a protective factor against depressed mood^18,19^ and the development of anxiety symptoms^20,21^. Individuals with high emotional intelligence could be better able to buffer the effects of stress to maintain and protect mental health^22^. In this context, abilities in emotion regulation appear to have a crucial role in controlling and reducing negative emotions such as anxiety^18^ and in promoting the development of positive emotions^17^. One strategy, among many others, to regulate one’s emotions is attentional deployment^23^. Attentional processes regulate emotional responses by tuning the filters for initial attention and subsequent information processing^24^. Common strategies of attention regulation refer to direct one’s attention to positive emotional information and to disengage one’s attention from or avoid negative emotional information^25^. Fiori^26^ suggested that individuals with high emotional intelligence might have a general preference for allocating attentional resources to emotional stimuli, which should facilitate their perception and recognition. However, trait emotional intelligence was not found to predict performance on facial emotion-processing tasks but was associated with a reduced stress response during the tests^27^.

To our knowledge, only two studies have investigated attention allocation to emotional information as a function of emotional intelligence using eye-tracking technology. Eye tracking has important methodological advantages in the assessment of attentional orientation compared to reaction time tests such as the dot-probe task^28^. Analysis of gaze behavior gives more direct and detailed evidence about, for example, which objects were attended for how long^29^. Lea et al.^30^ investigated the relationship of trait emotional intelligence with attention to positive emotional stimuli in a sample of healthy adults administering three passive viewing tasks with emotional faces and scenes as stimuli. Human faces have a special social significance^31^, they capture attention and are preferentially processed, in particular when emotions are expressed^32^. Lea et al.^30^ observed that emotional intelligence was associated with an attentional preference for positive stimuli, which they viewed as a factor that could promote mental health and function as protection from stressors.

A second eye-tracking study examining attention allocation to emotional information as a function of emotional intelligence and situational stress was conducted by Davis^33^. She used a dot probe task with a short stimulus presentation time (500 ms) to investigate early processes of attention allocation to facial emotions under stressful and neutral conditions and administered in addition to a trait measure two ability measures of emotional intelligence. In a sample of healthy individuals, sociability was found to be associated with a preference for looking first at angry rather than neutral faces across stress conditions. Under stress, well-being was related to an avoidance of sad faces. Additionally, analyses of attentional bias scores based on manual reaction times in the dot probe task suggested that emotion management ability went along with an avoidance of angry facial expressions. Interestingly, eye-movement bias scores were not related to bias scores based on manual RT in the study of Davis^33^. None of the emotional intelligence measures was associated with attention allocation towards or away from positive facial expression.

The divergent results between the two previous eye-tracking studies on attention and emotional intelligence^30,33^ could be due to methodological differences, in particular, the different tasks (dot probe vs. passive viewing), exposure or non-exposure to stressors, and the type of attention processes assessed (early and brief vs. late and sustained). Davis^33^ has examined early attentional preferences, whereas Lea et al.^30^ have investigated processes of sustained attention over a much longer time period. Overall, the current eye-tracking results suggest that trait emotional intelligence could be linked to an attentional preference of positive emotional stimuli at late stages of information processing.

Normal, healthy people show an attentional bias favoring positive-content stimuli, which is assumed to have a mood-protective function^34,35^. Despite a general preference for positive stimuli, there exist individual differences in healthy individuals’ attention deployment: high trait happiness goes along with enhanced sustained attention for different types of positive stimuli (achievement, social, and primary reward)^36^. Moreover, an attentional preference for positive facial expressions and stimuli was found to be linked to dispositional optimism^37^ and to more positive mood^38^. An attentional bias to positive information can prevent negative emotional responses to stressors and enhance reward perception^39,40^ and may constitute an adaptive part of antecedent emotion regulation that operates before a negative emotional response is generated via the elicitation of positive emotional experiences^41^.

Understanding how emotional intelligence operates to promote positive mood remains an important research question. The allocation of attentional resources towards positive information may be one pathway how emotional intelligence enhances positive affect and reduces negative affect. Specifically, when confronted with stimuli of different emotional valences, individuals with high emotion regulation ability may direct their visual attention towards positive or happy stimuli, which, in turn, could promote the activation of positive affect and prevent the activation of negative affect. In our study, we performed mediation analyses to assess the mediating effect of attention to happy faces on the relationship between emotion regulation abilities and trait affect. In our mediation models, intrapersonal and interpersonal emotion regulation abilities were used as independent variables in separate analyses. In all analyses, attention to happy faces was evaluated as mediator (i.e., dwell time on happy faces). Dependent variables were trait positive affect, trait negative affect, and trait anxiety. Trait affects represent relatively stable predispositions to certain (e.g., positive, or negative) emotional states. It was hypothesized that abilities of emotion regulation are positively correlated with heightened sustained attention to happy faces and with trait positive affect and negatively associated with trait negative affect and trait anxiety, respectively. In addition, we expected that attention to happy faces is related to high trait positive affect and low trait negative affect and low trait anxiety. In our analyses, we focused on attention to positive facial expressions since we utilized a free-viewing paradigm, which has shown to assess attentional preference for positive faces in healthy individuals in previous research from our laboratory^35,42^. In this task, four facial expressions (happy, sad, angry, and neutral) were shown simultaneously for ten seconds so that processes of sustained attention could be measured. A self-report instrument of trait emotional intelligence, the *Self-Rated Emotional Intelligence Scale* (SREIS^43,44^) was applied in the present study. The SREIS is based on Mayer and Salovey’s ability-based model of emotional intelligence and comprises the facets perception, use, understanding, and regulation of emotion^3^. Importantly, the facet emotion regulation is subdivided into the scales Managing emotion (self), which relates to the regulation of emotions in oneself, and Social management, which refers to the regulation of emotions in other persons. Given known associations of emotional intelligence as well as attentional biases with depressed mood^18,19,42,45^, we assessed study participants’ current level of depressive symptoms. Moreover, since the SREIS has been found to be positively associated with verbal (but not performance) IQ^46^ participants’ verbal intelligence was controlled in our study.

## Results

### Relations of emotional intelligence with positive and negative affectivity, trait anxiety, depression, and intelligence

Initially, we examined the correlations between the scales of the SREIS. Most of the scales correlated significantly with each other (see for details Supplementary Table S1). Exploratory analyses were calculated to examine gender differences on the scales of the SREIS (see for details Supplementary Table S2).

Next, we analyzed the relations of the SREIS scales with state and trait affectivity, trait anxiety, depression, and verbal intelligence. Managing emotion showed a positive correlation with trait positive affect, and moderate to high negative correlations with trait negative affect, trait anxiety, and depression (see Table 1). Social management was also positively related to trait positive affect, and negatively related to trait negative affect. State positive and state negative affect as well as verbal intelligence were not related to any of the SREIS scales. The SREIS total score was positively related to trait positive affect, and negatively related to trait negative affect, trait anxiety, and depression (see for details Table 1).

**Table 1:** Correlations of SREIS scales with state and trait affectivity (PANAS), trait anxiety (STAI), depression (BDI-II), and verbal intelligence (MWT-B) (N = 104).

	PE	UsE	UnE	ME	SM	Total
State positive affect	− 0.18	0.03	− 0.03	0.10	0.15	0.03
State negative affect	0.01	− 0.08	0.06	− 0.09	− 0.01	− 0.03
Trait positive affect	0.20*	0.14	0.33**	0.27**	0.35***	0.40***
Trait negative affect	− 0.17	− 0.10	− 0.08	− 0.43***	− 0.20*	− 0.30**
Trait anxiety	− 0.04	− 0.12	− 0.23*	− 0.54***	− 0.17	− 0.36***
Depression	0.02	− 0.12	− 0.05	− 0.32**	− 0.15	− 0.20*
Verbal intelligence	0.07	0.13	0.04	− 0.04	− 0.12	0.01

PE, perceiving emotion; UsE, use of emotion; UnE, understanding emotion; ME, managing emotion (self); SM, social management.

*p < 0.05, ** *p* < 0.01, *** *p* < 0.001.

### Dwell time as a function of facial expression

The analysis of variance with type of facial expression as within-subject factor revealed a significant difference in dwell times, *F*(3, 309) = 69.57, *p* < 0.001, *ƞ*^2^ = 0.403. The results of post-hoc pairwise comparisons indicated that participants looked longer at happy faces (M = 2824, SD = 659) than at neutral (M = 1963, SD = 350), sad (M = 2000, SD = 340), and angry faces (M = 2067, SD = 357) (*p*s < 0.001). Dwell times for neutral, sad, and angry facial expressions did not differ from one another (*p*s > 0.05). No significant gender differences were revealed for any of the dwell time scores (all *p*s > 0.05).

### Relations of emotional intelligence, positive and negative affectivity, trait anxiety, depression, and intelligence with sustained attention to emotional faces

The correlation analysis revealed that total emotional intelligence, managing emotion (self), social management, and trait positive affect (PANAS) were positively correlated with dwell time on happy faces. Trait anxiety (STAI) was negatively correlated with dwell time on happy faces. Moreover, total emotional intelligence and social management were negatively correlated with dwell time on angry faces. In contrast, state negative affect (PANAS) was positively correlated with dwell time on angry faces. There were no correlations of the SREIS scales with dwell times on sad or neutral faces. State positive affect, trait negative affect, depression, and verbal intelligence were not correlated with any of the dwell time scores (see for details Table 2).

**Table 2:** Correlations of sustained attention to facial expressions with facets of emotional intelligence (SREIS), state and trait affectivity (PANAS), trait anxiety (STAI), depression (BDI-II), and verbal intelligence (MWT-B) (N = 104).

	Dwell times			
	Happy faces	Angry faces	Sad faces	Neutral faces
SREIS total score	0.21*	− 0.20*	− 0.06	− 0.02
Perceiving emotion	− 0.03	− 0.07	0.11	0.03
Use of emotion	0.15	− 0.13	0.05	0.06
Understanding emotion	0.04	− 0.08	− 0.02	− 0.02
Managing emotion (self)	0.23*	− 0.15	− 0.16	− 0.06
Social management	0.27**	− 0.23*	− 0.13	− 0.06
State positive affect	0.11	0.06	− 0.15	− 0.09
State negative affect	0.01	0.23*	0.14	− 0.28**
Trait positive affect	0.25*	− 0.12	− 0.23*	− 0.17
Trait negative affect	− 0.16	0.17	0.02	0.04
Trait anxiety	− 0.24*	0.13	0.11	0.23*
Depression	− 0.10	0.16	− 0.06	0.16
Verbal intelligence	− 0.07	− 0.08	0.17	0.05

* *p* < 0.05, ** *p* < 0.01.

### Mediation analyses

**Figure 1:**
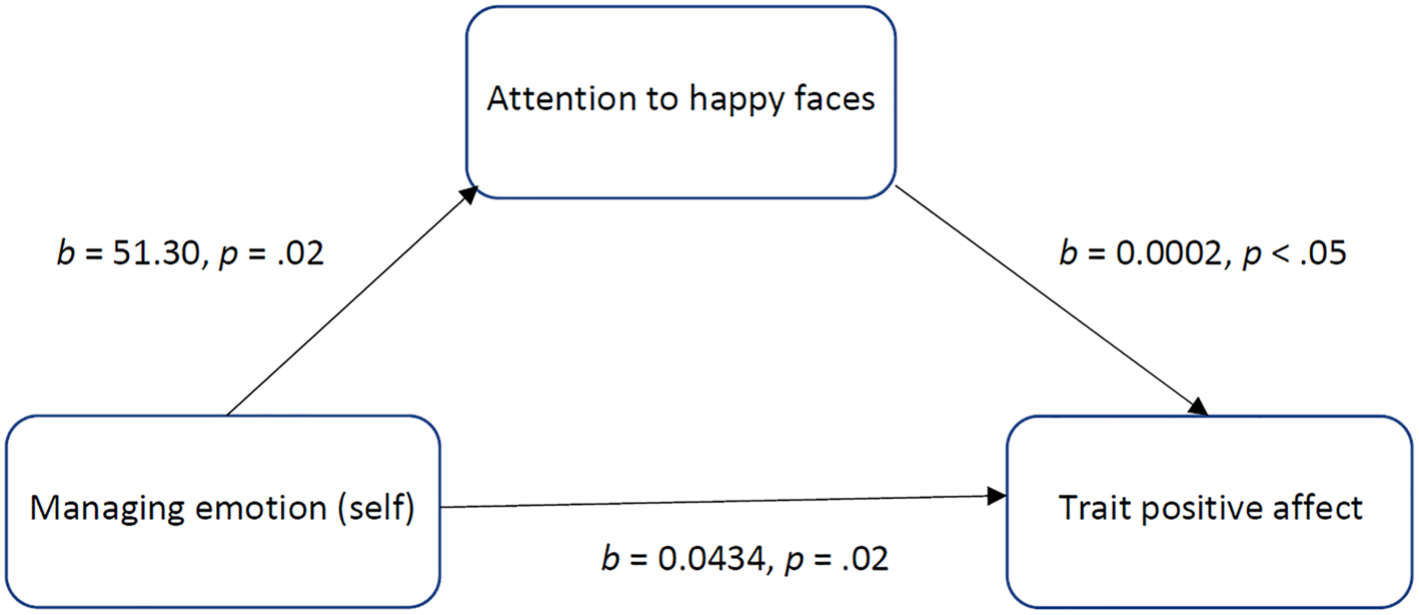
The mediating effect of attention to happy faces (dwell time on happy faces) in the relationship between managing emotion (self) and trait positive affect (PANAS). All presented effects are unstandardized.

**Figure 2:**
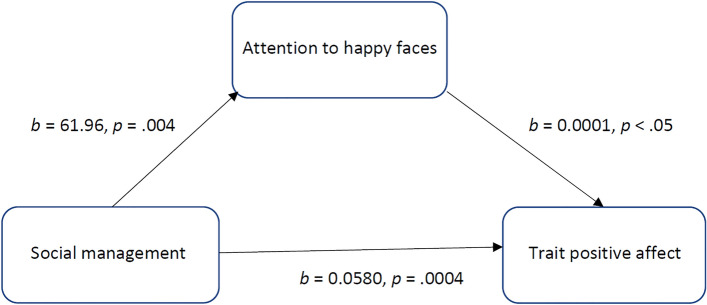
The mediating effect of attention to happy faces (dwell time on happy faces) in the relationship between social management and trait positive affect (PANAS). All presented effects are unstandardized.

Two models were tested to assess the mediating effect of attention to happy faces in the relationship of managing emotion (or social management) with trait positive affect as the dependent variable. The results of the first mediation analysis indicated that managing emotion was indirectly related to trait positive affect through its relationship with attention to happy faces. A 95% confidence interval based on 10,000 bootstrap samples showed that the indirect effect was entirely above zero (see Table 3). As can be seen in Fig. 1 managing emotion was a positive predictor of attention to happy faces and attention to happy faces was a positive predictor of trait positive affect. There was also evidence of a direct effect of managing emotion on trait positive affect, independent of attention to happy faces (see Table 3). The results of the second mediation model suggested that social management was indirectly related to trait positive affect through attention to happy faces. As can be seen in Table 4 the confidence intervals for the indirect effect did not cross zero. Social management was a positive predictor of attention to happy faces and attention to happy faces was a positive predictor of trait positive affect (see Fig. 2). Moreover, social management had also a direct effect on trait positive affect, independent of attention to happy faces (see Table 4).

**Table 3:** Mediation model for the effect of managing emotion (self) on trait positive affect (PANAS) via attention to happy faces (dwell time on happy faces).

Effect type	Paths	Effect	SE	*t*	LLCI	ULC**I**
Direct	ME → trait positive affect	0.0434	0.0187	2.32*	0.0063	0.0804
Indirect	ME → attention to happy faces → trait positive affect	0.0085	0.0054		0.0008	0.0216
Total	ME → trait positive affect	0.0519	0.0185	2.81**	0.0153	0.0885

ME, managing emotion (self); SE, standard error; LLCI, lower limit confidence interval; ULCI, upper limit confidence interval

* *p* < 0.05, ** *p* < 0.01.

**Table 4:** Mediation model for the effect of social management on trait positive affect (PANAS) via attention to happy faces (dwell time on happy faces).

Effect type	Paths	Effect	SE	*t*	LLCI	ULC**I**
Direct	SM → trait positive affect	0.0580	0.0157	3.69*	0.0268	0.0892
Indirect	SM → attention to happy faces → trait positive affect	0.0086	0.0049		0.0001	0.0191
Total	SM → trait positive affect	0.0666	0.0154	4.34*	0.0362	0.0971

The HC3 (Davidson-MacKinnon) procedure for heteroscedasticity-consistent inference was used in this analysis.

SM, social management; SE, standard error; LLCI, lower limit confidence interval; ULCI, upper limit confidence interval

* *p* < 0.001.

Two further mediation analyses were performed to assess the mediating effect of attention to happy faces in the relationship of managing emotion (or social management) with trait negative affect as the dependent variable. The results of both mediation analyses indicated no mediating effects of attention to happy faces on the association between managing emotion (or social management) and trait negative affect (see Tables S3 and S4 in the Supplementary information).

**Figure 3:**
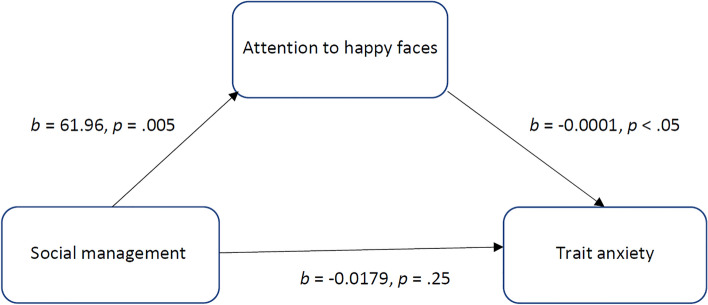
The mediating effect of attention to happy faces (dwell time on happy faces) in the relationship between social management and trait anxiety (STAI). All presented effects are unstandardized.

Finally, two models were tested to assess the mediating effect of attention to happy faces in the relationship of managing emotion (or social management) with trait anxiety as the outcome variable. The results of the first mediation analysis indicated that attention to happy faces had no mediating effect on the relationship between managing emotion and trait anxiety (see for details Supplementary Table S5). The results of the second mediation model indicated that social management was indirectly related to trait anxiety through attention to happy faces. As can be seen in Table 5 the confidence intervals for the indirect effect did not cross zero. Social management was a positive predictor of attention to happy faces, which in turn negatively predicted trait anxiety (see Fig. 3). Social management had no direct effect on trait anxiety (see Table 5). Thus, the relationship between social management and trait anxiety was fully mediated by attention to happy faces.

**Table 5:** Mediation model for the effect of social management on trait anxiety (STAI) via attention to happy faces (dwell time on happy faces).

Effect type	Paths	Effect	SE	*t*	LLCI	ULC**I**
Direct	SM → trait anxiety	− 0.0179	0.0153	− 1.17	− 0.0484	0.0125
Indirect	SM → attention to happy faces → trait anxiety	− 0.0087	0.0051		− 0.0201	− 0.0005
Total	SM → trait anxiety	− 0.0266	0.0150	− 1.77	− 0.0563	0.0031

SM, social management; SE, standard error; LLCI, lower limit confidence interval; ULCI, upper limit confidence interval.

## Discussion

Analyzing how emotional intelligence relates to attentional preferences as assessed by gaze behavior remains a poorly explored area of research. The allocation of attentional resources towards positive information may protect and enhance mood and well-being. To our knowledge, the present eye-tracking study represents the first to examine the mediating effect of attention to happy faces on the relationship between emotion regulation abilities and affect. The results of our mediation analyses suggest that a positive attentional bias might be a cognitive mechanism, by which the association of intrapersonal and interpersonal emotion regulation with trait positive affect can be explained, at least in part. Directing one’s attention preferentially to positive information could act as one mechanism, by which emotion regulation abilities may activate positive affect in everyday life. A central role in inducing positive affective reactions during the perception of happy facial expressions could play processes of emotional contagion. Emotional contagion refers to a phenomenon of an automatic adoption of an emotional state of another person^47^ and is assumed to occur through facial mimicry and feedback^48^. The receiver imitates the sender’s emotional display in emotional mimicry. Then, facial feedback from such mimicry elicits the corresponding emotional state in the receiver. The perception of happy facial expressions elicits happy feelings in the receiver through facial muscle activity^49^. In summary, it can be concluded that abilities in intrapersonal and interpersonal emotion regulation may both heighten attention for positive social information, which could favor the activation of positive emotions. The ability to regulate other people’s emotions and to enhance their mood seems to have a positive impact on one’s own mood. Our results are basically consistent with those of Lea et al.’s study^30^, which has examined the relationship between trait emotional intelligence and late attentional preferences. The authors reported positive associations of global trait emotional intelligence and the factors self-control, emotionality, and well-being with fixation time on happy faces.

As expected, we found that intra- as well as interpersonal emotion regulation abilities were associated with trait positive affect. Previous research has shown that emotional intelligence goes along with happiness and optimism^16,17^ and that abilities in emotion regulation appear to play an important role in promoting the development of positive affects^17^. Positive emotions can be induced or enhanced in various other ways than attention allocation: for example, by engaging in activities that are pleasurable^50^ or by recalling positive personal experiences^51^.

According to our mediation analyses, no mediating effects of attention to happy faces were observed for the relationships of managing emotion and social management with trait negative affect, although managing emotion and social management showed, as hypothesized, significant negative correlations with trait negative affect. Contrary to expectation, attention to happy faces was not associated with trait negative affect. Similarly, we revealed no mediating effect of attention to happy faces on the relationship between managing emotion in the self and trait anxiety. However, social management was found to be indirectly related to trait anxiety through attention to happy faces. As social management had no direct effect on trait anxiety, the relationship between social management and trait anxiety was fully mediated by attention to happy faces. Allocating one’s attention preferentially to positive information (which may imply in our free viewing task also an averting of attention from negative and threatening expressions) could represent a mechanism, by which interpersonal emotion regulation ability reduces the occurrence of anxiety reactions in daily life. In this context, it should be noted that in our study interpersonal emotion regulation was also found to be negatively correlated with dwell time on angry faces. That is, high ability in interpersonal emotion regulation was connected to an attentional avoidance of threatening facial expressions. Habitually paying more attention to positive instead of threat-related social signals could help to reduce processing of anxiety-producing stimuli. Previous research on stress and resilience has revealed that attention allocation to positive information can enhance reward perception and prevent negative emotional responses to stressors^39,40^. An attentional preference for positive stimuli can be a part of antecedent emotion regulation that operates before negative responses such as anxiety develop via the generation of positive emotions^41^. Individuals high in emotional intelligence seem to use more antecedent-focused strategies of emotion regulation^52^. An attention style characterized by attentional avoidance of threatening stimuli at low levels of danger is considered as adaptive and promoting mental health^53,54^.

In our free-viewing task, we presented four facial expressions simultaneously (happy, sad, angry, and neutral faces), which competed for attention. Human faces are stimuli of high social significance, which, in general, capture attention and are preferentially processed^31,32^. The analysis of dwell times revealed that, on average, our healthy participants viewed substantially longer at happy than at sad, angry, or neutral faces. This finding is consistent with previous research showing an attentional bias favoring positive-content stimuli in healthy people^42,55,56^. Considering neutral faces as baseline condition, we observed clear evidence for an attentional preference of happy faces but not for an avoidance of negative facial expressions in our task. In future studies on the topic, stimulus pairs composed of a neutral and an emotional face could be used. In this way, attentional orientation towards positive and away from negative expressions can be separately assessed and analyzed.

It is noteworthy that perceiving emotion and use of emotion, which form the two facets of experiential emotional intelligence, were not or hardly correlated with any of the affectivity scales. In addition, these components of emotional intelligence (as well as the facet understanding of emotions) were not associated with attention allocation to emotion stimuli in our sample. This supports the idea that it is not experiential emotional intelligence but a specific facet of strategic emotional intelligence, i.e., regulation of emotion, that might be primarily responsible for attention allocation during the perception of emotional information.

Emotional intelligence can be increased through training and such interventions have the potential to lead to improved mental and physical health, and relationship satisfaction^57^. The role of emotion regulation capabilities in the deployment of attention could be further clarified in future intervention studies examining changes in attention allocation to emotional stimuli as a function of improved regulation abilities. The link between affect and attention is assumed to be bidirectional: affect influences deployment of attention but regulating attention also can have an impact on affect and affective cognition^25^. Attention bias modification (ABM) trainings have been developed that offer computer-delivered treatment for anxiety disorders^58^ and depressive disorders^59^. These ABM training methods intend to reduce attentional biases to threatening or dysphoric information by reducing excessive attentional allocation to negative stimuli. Another ABM method is positive-search training, which encourages participants to search for positive target cues. Such positive-search trainings have been administered to increase optimism regarding future positive events^60^. It is an interesting question whether trait emotional intelligence or emotion regulation abilities can be augmented by ABM trainings.

In sum, the results of our eye-tracking study suggest an association of emotion regulation abilities with an attentional preference for positive social signals under non-stressful conditions. Preference of positive social information could form one attentional pathway through which emotion management abilities enhance mood and buffer the effects of threats. However, it is important to note that our study was cross-sectional, and no causal inferences can be made from the present results. Further research is needed to investigate how abilities in emotion regulation affect visual processing under stressful, threatening conditions and after sad mood induction. If emotion management abilities are adaptive, they may facilitate shifts towards preference for threat stimuli in stressful situations and increase preferences for positive stimuli following inductions of sad mood. For a more comprehensive investigation of how and what facets of emotional intelligence influence attention allocation to emotional information it might be useful to administer performance-based ability measures in addition to self-report questionnaires in future studies using comprehensive instruments of both constructs.

## Materials and methods

### Participants

Our sample consisted of 104 healthy young individuals (71 women) with a mean age of 24.02 years (*SD* = 4.22, range = 18–35). The mean duration of participants’ school education was 12.19 years (SD = 1.01). 81.8% of the sample were university students from various disciplines (n = 76) or apprentices (n = 9). All participants were native German speakers. They were screened for multiple exclusion criteria, pregnancy, compromised vision, current or previous psychiatric or neurological disorders, and abuse of alcohol or other substances before taking part in the study. All participants gave written informed consent before inclusion in the study. Participants were recruited via public and online notices at the University of Leipzig. The study was conducted in accordance with the Declaration of Helsinki^61^. The ethics committee of the University of Leipzig, Medical School, approved our study (reference number 186/14-ek).

### Self-report questionnaires

#### Emotional intelligence: Self-Rated Emotional Intelligence Scale

The German version of the *Self-Rated Emotional Intelligence Scale* (SREIS^43^) was administered, which assesses four facets of emotional intelligence, i.e., perception, use, understanding, and regulation of emotion^44^. The SREIS is a self-report measure, based on Mayer and Salovey’s ability-based model of emotional intelligence^1^. The SREIS comprises five subscales as the facet “emotion regulation” is subdivided into the regulation of emotions in oneself (*Managing emotion (self)*) and regulation of emotions in other persons (*Social management*). The scale Perceiving emotion includes four items related to the perception and accurate recognition of emotions expressed by other persons (e.g., “I am aware of the nonverbal messages other people send”). The scale Use of emotion comprises three items related to the ability to harness feelings that assist in cognitive processes such as reasoning, problem solving, and interpersonal communication (e.g., “When making decisions, I listen to my feelings to see if the decision feels right”). The scale Understanding emotion consists of four items, which refer to the emotional lexicon, the ability to understand and verbally describe feelings (e.g., “I could easily write a lot of synonyms for emotion words like happiness or sadness”). The scale Managing emotion (self) includes four items referring to the ability to reduce, or modify emotional responses in oneself (e.g., “I know how to keep calm in difficult or stressful situations”), and the scale Social management comprises four items relating to the ability to regulate other persons’ emotions (e.g., “I know the strategies to make or improve other people’s moods”). A total SREIS score can be calculated by summing the subscale scores. Participants score each item of the SREIS on a Likert-type scale ranging from 1 (inaccurate) to 5 (accurate). In our study, Cronbach’s alpha was 0.65 for Perceiving emotion, 0.81 for Use of emotion, 0.86 for Understanding emotion, 0.71 for Managing emotion (self), 0.76 for Social management, and 0.84 for the total SREIS score. Thus, internal consistencies were satisfactory for the SREIS scales with the exception of Perceiving emotion.

#### State and trait affect: Positive and Negative Affect Schedule

State and trait positive and negative affects were assessed with the *Positive and Negative Affect Schedule* (PANAS^62^). The PANAS consists of 10 negative and 10 positive adjectives, which are rated on a five-point Likert scale (from 1 to 5). In the state version of the PANAS, participants are asked how they feel at the present moment, whereas in the trait version, they are asked to describe how they feel in general. In the present sample, Cronbach’s alpha was 0.80 for state positive affect, and 0.83 for state negative affect. Moreover, Cronbach’s alpha was 0.83 for trait positive affect, and 0.81 for trait negative affect. In our sample, the mean state positive affect item score was 2.86 (SD = 0.59) and the mean state negative affect item score was 1.46 (SD = 0.47), whereas the mean trait positive affect item score was 3.33 (SD = 0.56) and the mean trait negative affect item score was 1.58 (SD = 0.46).

#### Depression: Beck Depression Inventory

The *Beck Depression Inventory* (BDI-II^63^) measures the severity of depressive symptoms experienced during the last two weeks. The BDI-II is composed of 21 items that relate to symptoms such as hopelessness, suicidal ideation, and negative cognitions. Each item of the BDI-II has four response options that range from 0 to 3. In our sample, Cronbach’s alpha was 0.77 for the BDI-II. In the present study, the mean BDI-II score was 8.85 (SD = 5.15). Our mean BDI-II score is similar to that found in a large German sample of healthy adults (M = 7.69, SD = 7.52)^64^.

#### Anxiety: State and Trait Anxiety Inventory

The trait version of the *State and Trait Anxiety Inventory* (STAI^65^) was used to assess participants’ dispositional anxiety. The STAI consists of 20 items. The items of the STAI trait version are rated on a 4-point scale (from 1, “almost never”, to 4, “almost always”). Cronbach’s alpha for trait anxiety was 0.89. In our study, the mean STAI trait anxiety score was 38.46 (SD = 8.95).

### Measure of verbal intelligence: Multiple-Choice Vocabulary Test

Participants’ verbal intelligence was evaluated by the *Multiple-Choice Vocabulary Test* (*Mehrfachwahl-Wortschatz-Intelligenztest)* version B (MWT-B^66^). The MWT-B includes 37 items and has no time restrictions. Each item comprises one real word and four pronounceable pseudo-words (fictitious words). Subjects have the task to mark the real word. The raw scores (number of correct answers) can be transformed into IQ scores. In healthy adults, the MWT is highly correlated with global IQ^67^. In our sample, the mean IQ score was 109.32 (SD = 11.50).

### Free viewing task: facial stimuli and procedure

A free viewing task was used to assess attention to emotional faces. 80 photographs of 20 actors were selected from the Lifespan Database of Adult Emotional Facial Stimuli^68^. Each actor expressed three emotional states: happiness, sadness, and anger. In addition, photographs of each actor with a neutral facial expression were included. The four facial expressions of an actor were arrayed in a 2 × 2 matrix and simultaneously presented on the screen (see^35,42^ for examples of stimuli). The display size of each facial expression was 13 × 11 cm (height by width). The vertical distance between the centers of the photographs was 14 cm, whereas the horizontal distance was 12 cm. The pictures were displayed in their original color against a white background on a 22-in. TFT widescreen monitor with a resolution of 1680 × 1050. The free viewing task consisted of 20 trials.

### Eye-tracking: apparatus and eye movement parameter

Gaze behavior was recorded using an IView X RED250 remote eye-tracker system by SensoMotoric Instrumens (SMI), an infrared video-based eye-tracking device recording eye movements every 4 ms (250 Hz) with a gaze position accuracy of 0.4°. Fixations were defined as stable gaze locations within a 1° radius of visual angle with a minimum duration of 100 ms. A velocity-based algorithm with a minimum fixation duration of 100 ms, a minimum saccade duration of 22 ms and a peak velocity threshold of 40°/s was used to record the eye-tracking data. No head-resting device was required since the SMI RED250 tracking system is capable of compensating changes in head position. A SMI-customized Dell laptop (IView X) was used for stimulus presentation and data acquisition. The software SMI Experiment Center controlled presentation of stimuli and synchronization with recorded eye movements.

SMI BeGaze 3.0 software was utilized to define four areas of interest (AOIs) in each trial corresponding to each face image (happy, neutral, sad, and angry). We were interested in measuring the time participants looked at each AOI. Dwell time was used as an indicator of sustained attention. Dwell time was defined as the sum of durations (in milliseconds) of all fixations and saccades on the area of interest, i.e., the neutral or the emotional facial expressions. Dwell times were computed for each facial expression during each trial and then averaged for every participant. Eye tracking data were pre-processed with SMI BeGaze 3.0.

### General procedure

The experiment took place at the Department of Psychosomatic Medicine and Psychotherapy at the University of Leipzig. Study participants were invited to individual laboratory sessions. The experiment was conducted in a quiet room shielded from sunlight. Ceiling lights ensured stable lighting conditions. After the eye-tracking experiment, participants completed the PANAS state, the BDI-II, the STAI, the SREIS, the MWT-B and the PANAS trait. Participants received a fee of 30 € for taking part in the experiment.

### Statistical analysis

Pearson correlation analysis was performed to investigate the linear relationships between facets of emotional intelligence, state and trait affectivity, trait anxiety, depression, intelligence, and attention allocation to facial expressions. To explore gender differences in emotional intelligence as well as attention processes in the free viewing task *t*-tests for independent samples were administered. A repeated measure ANOVA (followed by post-hoc pairwise comparisons) was conducted on dwell times to test for differences in sustained attention between the four expression conditions (happy, neutral, sad, and angry). Results were considered significant at *p* < 0.05, two-tailed. Statistical calculations were made with SPSS 27.0 (IBM Corp., Armonk, NY, USA).

Mediation analyses were performed with the PROCESS macro for SPSS applying Model 4^69^. Bias-corrected bootstrap analyses based on 10,000 bootstrap samples were run. The bias level was set to 95%. We conducted the modified Breusch-Pagan test to assess heteroscedasticity of the residuals from the linear regressions. In case of detection of heteroscedasticity, we used the HC3 (Davidson-MacKinnon) procedure for heteroscedasticity-consistent inference in the PROCESS options. Indices of the indirect effect were considered statistically significant if the 95% CI, estimated using bootstrap method, did not include zero. In the mediation models, intrapersonal and interpersonal emotion regulation, i.e., managing emotion (self) and social management, were separately used as independent variables in different analyses. Attention to happy faces was evaluated as mediator (i.e., dwell time on happy faces). Dependent variables were trait positive affect, trait negative affect, and trait anxiety.

## Supplementary Information

Supplementary Tables.

## Data Availability

The datasets used and analyzed during the current study are available from the corresponding author on reasonable request.
