# Supplementary material for: Positive attentional bias mediates the relationship between trait emotional intelligence and trait affect
Source: Sci Rep. 2022 Dec 1;12:20733. doi: 10.1038/s41598-022-25317-9 (PMC9715682; doi:10.1038/s41598-022-25317-9)
Supplement: Supplementary file 1 — Supplementary Tables. [file 41598_2022_25317_MOESM1_ESM.pdf]

Supplementary information

Positive attentional bias mediates the relationship between trait emotional intelligence and trait affect

Thomas Suslow, Dennis Hoepfel, Vivien Günther, Anette Kersting, Charlott Maria Bodenschatz

Department of Psychosomatic Medicine and Psychotherapy, University of Leipzig Medical Center, Semmelweisstr. 10, 04103 Leipzig, Germany.

Corresponding author: Thomas Suslow, suslow@medizin.uni-leipzig.de

Table S1. Descriptive statistics of and correlations between SREIS scales (N = 104).

**SREIS scales**	**UsE**	**UnE**	**ME**	**SM**	**Total**	Mean	SD
Perceiving emotion	.24*	.43***	.03	.40***	.60***	15.86	2.21
Use of emotion		.37***	-.01	.25*	.57***	10.55	2.79
Understanding emotion			.23*	.42***	.79***	12.97	3.45
Managing emotion (self)				.31**	.50***	13.33	2.90
Social management					.74***	14.81	2.91
Total SREIS score						67.51	9.25

* $p < .05$, ** $p < .01$, *** $p < .001$
UsE: Use of Emotion; UnE: Understanding Emotion; ME: Managing Emotion (self); SM: Social Management.

Table S2. Scores on the SREIS scales (means and SDs) as a function of gender.

	Women (n=71)		Men (n=33)		
	Mean	SD	Mean	SD	*t (102)*
SREIS total score	69.34**	9.03	63.58**	8.60	3.01*
Perceiving emotion	16.31**	2.05	14.88**	2.26	3.21*
Use of emotion	11.18**	2.65	9.18**	2.62	3.60**
Understanding emotion	13.58*	3.40	11.67*	3.23	2.71*
Managing emotion (self)	13.17	3.04	13.67	2.57	-0.81
Social management	15.10	2.90	14.18	2.88	1.50

Significant differences according to *t*-tests for independent samples: * $p < .01$, ** $p < .001$

Table S3. Mediation model for the effect of managing emotion (self) on trait negative affect (PANAS) via attention to happy faces (dwell time on happy faces). The HC3 (Davidson-MacKinnon) procedure for heteroscedasticity-consistent inference was used in this analysis.

**Effect type**	**Paths**	**Effect**	**SE**	***t***	**LLCI**	**ULCI**
Direct	ME ⇨ trait negative affect	-.0645	.0141	-4.57*	-.0925	-.0365
Indirect	ME ⇨ attention to happy faces ⇨ trait negative affect	-.0025	.0038		-.0110	.0044
Total	ME ⇨ trait negative affect	-.0670	.0130	-5.17*	-.0927	-.0413

ME: Managing emotion (self), SE: standard error, LLCI: Lower limit confidence interval, ULCI; Upper limit confidence interval, * $p < .001$.

Table S4. Mediation model for the effect of social management on trait negative affect (PANAS) via attention to happy faces (dwell time on happy faces).

**Effect type**	**Paths**	**Effect**	**SE**	***t***	**LLCI**	**ULCI**
Direct	SM ⇨ trait negative affect	-.0266	.0157	-1.69	-.0578	.0046
Indirect	SM ⇨ attention to happy faces ⇨ trait negative affect	-.0050	.0050		-.0163	.0028
Total	SM ⇨ trait negative affect	-.0316	.0152	-2.08*	-.0616	-.0015

SM: Social management, SE: standard error, LLCI: Lower limit confidence interval, ULCI; Upper limit confidence interval, * $p < .05$.

Table S5. Mediation model for the effect of managing emotion on trait anxiety (STAI) via attention to happy faces (dwell time on happy faces).

Effect type	Paths	Effect	SE	*t*	LLCI	ULCI
Direct	ME ⇨ trait anxiety	-.0794	.0131	-6.04*	-.1055	-.0533
Indirect	ME ⇨ attention to happy faces ⇨ trait anxiety	-.0042	.0038		-.0135	.0010
Total	ME ⇨ trait anxiety	-.0836	.0129	-6.50*	-.1091	-.0581

ME: Managing emotion (self), SE: standard error, LLCI: Lower limit confidence interval, ULCI; Upper limit confidence interval, * $p < .001$.
